# New Findings on Multilayer Silicene on Si(111)√3×√3R30°–Ag Template

**DOI:** 10.3390/ma12142258

**Published:** 2019-07-13

**Authors:** Paola De Padova, Amanda Generosi, Barbara Paci, Carlo Ottaviani, Claudio Quaresima, Bruno Olivieri, Marek Kopciuszyński, Lucyna Żurawek, Ryszard Zdyb, Mariusz Krawiec

**Affiliations:** 1 Consiglio Nazionale delle Ricerche-ISM, Via Fosso del Cavaliere 100, 00133 Roma, Italy; 2 INFN-Laboratori Nazionali di Frascati Via Enrico Fermi 40, Frascati, 00044 Roma, Italy; 3 Consiglio Nazionale delle Ricerche-ISAC, Via Fosso del Cavaliere 100, 00133 Roma, Italy; 4 Institute of Physics, Maria Curie-Sklodowska University, pl. M. Curie-Sklodowskiej 1, 20-031 Lublin, Poland

**Keywords:** multilayer silicene, LEED/RHEED, ED-GIXRD, GWAXS/GSAXS, ARPES, DFT

## Abstract

We report new findings on multilayer silicene grown on Si(111)√3 × √3 R30°–Ag template, after the recent first compelling experimental evidence of its synthesis. Low-energy electron diffraction, reflection high-energy electron diffraction, and energy-dispersive grazing incidence X-ray diffraction measurements were performed to show up the fingerprints of √3 × √3 multilayer silicene. Angle-resolved photoemission spectroscopy displayed new features in the second surface Brillouin zone, attributed to the multilayer silicene on Si(111)√3 × √3 R30°–Ag. Band-structure dispersion theoretical calculations performed on a model of three honeycomb stacked layers, silicene grown on Si(111)√3 × √3 R30°-Ag surface confirm the experimental results.

## 1. Introduction

The first synthesis of multilayer silicene on Si(111)√3 × √3 R30°–Ag interface used as a template was recently reported [1]. Experimental results, mainly including the structural and electronic properties investigated through Auger electron spectroscopy, low-energy electron diffraction (LEED), scanning tunneling microscopy/spectroscopy (STM/STS), in situ Raman spectroscopy, energy-dispersive (ED) grazing incidence X-ray diffraction (GIXRD), and angle-resolved photoelectron spectroscopy (ARPES) in communion with the density functional theory (DFT) calculations prove the possibility of obtaining silicene beyond the first layer and almost directly grown on silicon substrate [1].

If on one hand the X-ray diffraction results give the size of the new √3 × √3 elementary cell of the silicene superstructure, on the other, the results of the theoretical calculations provide for the first, second, and third layers of stacked silicene an alternative structure to that of diamond-type for silicon [1].

The first silicene layer is considered as free-standing, whereas the succession of bilayer and three-layer silicene on Si(111)√3 × √3 R30°–Ag surface are stacked with an AA’- and AA’B-type structure, following a relative minimum energy in close agreement with all the experimental findings [1].

The concept of a metastable system holds both from the experimental and the theoretical point of view for the delicate preparation of the samples, whose growth parameters, above all the temperature of the substrate and the flux of the silicon evaporation source, reside in a quite narrow window. This led to a spread of results that fueled the debate on the possible nonexistence of the silicene multilayer, as composed of stacked layers with internal honeycomb structure [2,3,4,5,6,7,8].

In this paper, we report new structural results using ED-GIXRD at small as well as wide angle, reflection high-energy electron diffraction (RHEED), ARPES, and band structures theoretical calculations to corroborate the evidence of multilayer silicene obtained from epitaxial growth upon Si(111)√3 × √3 R30°–Ag template.

## 2. Materials and Methods 

Sharp 7 × 7 reconstruction of Si(111) surface was obtained after several cycles of substrate flashing at ~1000 °C, for several times under ultra-high-vacuum conditions (base pressure 0.8 × 10^−10^ mbar). The Si(111)√3 × √3 R30°–Ag template was produced both by depositing one monolayer (ML) of silver from a W crucible at a rate of 0.05 ML/min onto the clean Si(111)7 × 7 crystal at ~500 °C [9,10], and by evaporation of Ag (~1 ML) at room temperature (RT) on Si(111)7 × 7, followed by annealing at about 500 °C for 5 min [9]. Silicon was deposited at a ~0.02 ÷ 0.05 ML/min rate from a source consisting of a directly heated Si-wafer piece, keeping the Si(111)√3 × √3 R30°–Ag sample at two temperatures of 200 °C and 250 °C. All surface reconstructions obtained from Si(111)7 × 7, Si(111)√3 × √3 R30°–Ag and Si-√3 × √3 film on Si(111)√3 × √3 R30°–Ag were observed by low-energy electron diffraction (LEED).

Angle-resolved photoemission spectroscopy measurements were performed by using unpolarized He Iα radiation (hν = 21.2 eV) on Si(111)√3 × √3 R30°–Ag interface and on 4 MLs Si grown on Si(111)√3 × √3 R30°–Ag template at temperatures of ~200 °C and ~250 °C. The photoemission spectra were acquired at room temperature (RT) by using an angular-resolved mode of the commercial hemispherical electron energy analyser (SPECS Phoibos 150, SPECS GmbH, Berlin, Germany), with an acceptance angle of ~14°. The band dispersions were collected in the second surface Brillouin zone (SBZ) at the high-symmetry point $\bar{\Gamma_{1}}$ along the [11$\bar{2}$] direction. In situ RHEED patterns were collected at RT (Ep = 18.6 keV) with electron beam along the [11$\bar{2}$] on each sample preparation. 

ED diffraction measurements in reflection mode were performed ex situ, fixing the instrumental geometry at ϑ = 5° and impinging the films with an X-ray beam, produced by a W-anode tube up to 55 keV. Detection was accomplished by an EG&G Solid State Detector (SSD, EG&G, Idaho Falls, ID, USA), whose sensitive part is a semiconducting high-purity germanium single crystal. This crystal is doped by another suitable element (lithium) in order to create a p–n junction inside it, and makes it behave like a diode that, in working conditions, is directly polarized by applying a high voltage at its sides. The SSD was connected via ADACAM hardware to a personal computer running the Maestro software to visualize and record the data through a multi-channels analyzer. In this way, a digital reconstruction of the energy spectrum of the scattered radiation accomplished.

Band-structure dispersion calculations were extracted from the DP4 theoretical model [1] for √3 × √3 superstructure of three-layer silicene on Si(111)√3 × √3 R30°–Ag, obtained by applying the density functional theory within the Perdew–Burke–Ernzerhof (PBE) [11] generalized gradient approximation (GGA) using projector-augmented wave potentials, as implemented in VASP (Vienna ab-initio simulation package) [12,13]. The Brillouin zone (BZ) sampled was 5 × 5 × 1 Monkhorst–Pack k-points grid [14] and the plane-wave energy cut off at 340 eV, as extensively specified in Ref. [1]. 

## 3. Results and Discussion

Multilayer silicene [15,16,17,18,19,20,21,22,23,24,25,26,27,28,29] was synthesized by depositing 10 MLs of silicon at ~200 °C on top of Si(111)√3 × √3 R30°–Ag (√3 × √3 R30°–Ag, hereafter called √3-Ag) interface.

**Figure 1:**
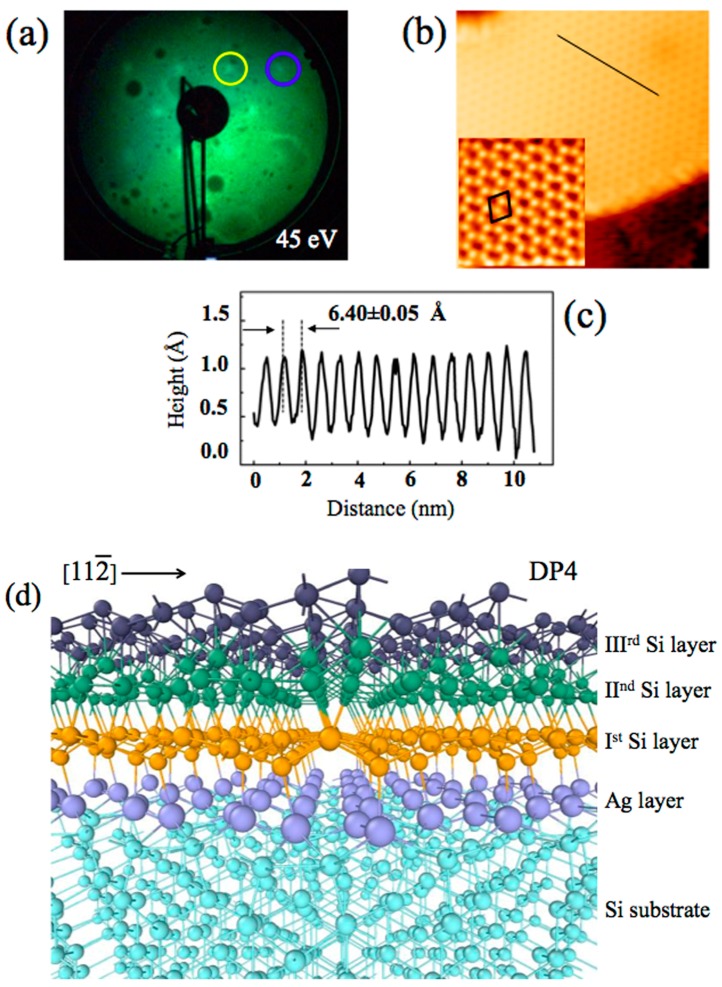
LEED patterns for ~10 MLs of silicon deposited onto the Si(111)√3–Ag surface at ~200 °C, yellow circle: (1/3,1/3) order spot of the Ag √3 × √3 reconstruction; blue circle: silicon integer order spot (**a**). Empty- and filled-state STM constant-current images, measured at 77 K from ~5 MLs of silicon deposited onto Si(111)√3–Ag surface at ~200 °C: (**b**) 15 × 15 nm^2^ (50 pA and −1.0 V) and its inset 5 × 5 nm^2^, (50 pA and 0.4 V) at atomic resolution; √3 × √3-Si unit cell is drawn. (**c**) is the line profile of the black line of (b). (**d**) is the side view three-dimensional (3D) representation of the DP4 model. Adapted with permission from (J. Phys. Chem. C, **2017**, 121, 27182–27190). Copyright (2017) American Chemical Society.

The √3 × √3 reconstruction of the multilayer silicene, with respect to the 1 × 1 silicene, is presented in Figure 1a by LEED pattern, where blue circle is the silicon integer-order spot of (1 × 1) silicene and the yellow (1/3,1/3) that of √3 × √3 reconstruction [1].

Scanning tunneling microscopy (STM) images reported in Figure 1b were acquired by using a low-temperature ultra-high-vacuum (UHV) scanning tunneling microscopy/scanning near-field optical microscopy system (LT-STM-SNOM) (SNOM1400, Unisoku Co. Ltd., Osaka, Japan) at 77 K, in constant current mode [1], showing a terraced film [15,16,17,22,23,24,25,26,27,29,30].

Empty- and filled-state STM at 15 × 15 nm^2^ (50 pA and −1.0 V) and 5 × 5 nm^2^ (50 pA and 0.4 V) atomic resolution images show the √3 × √3 multilayer silicene (unit cell is drawn) and (c), which is the line profile of the black line traced in (b) that clearly quantifies the side of the √3 × √3 reconstruction as 6.40 Å, in nice agreement with references [16,23,24]. The three-dimensional representation side view, along the [11$\bar{2}$] direction, of the DP4 model [1] is reported in (d). 

To scrutinize the crystallographic structure of materials, X-ray diffraction is the most widely used technique. The basic principle behind all investigations is the solution of Bragg’s equation, 2.d_hkl_.sinϑ = n.λ. Where the energy-dispersive X-ray diffraction is used, the Bragg angle (ϑ) is kept constant during measurements, the value of the lattice plane distance (d_hkl_) in Bragg’s equation being obtained via the experimental determination of the polychromatic radiation diffracted beam wavelength (λ) [31]. Indeed, when ED experiments are performed, a continuous spectrum radiation—typically the Bremmstrahlung of an X-ray tube, often called white by analogy with the visible light—is used as probe and the scattering angle remains fixed [31]. The detection is accomplished by an SSD, probing the incoming photons and their energy. The main drawback related to the ED mode is the reduced q resolution, due to the contribution of the SSD energy resolution, enlarging the diffraction peaks. However, when, as in the present case, ex situ measurements are performed and the same scattering volume must be sampled, high precision is gained despite the lower resolution, and different diffraction patterns can be easily compared in order to observe small variations.

Among the advantages of this technique, the ED method gains data over a larger q range (q = (4π.sin ϑ)/λ = 2π/d_hkl_ [Å^−1^]), giving better real-space structural resolution, which is crucial in our experiment. Indeed, the inverse relation between X-ray energy and d_hkl_ implies that when X-rays of high energy are available (for instance, up to 55 keV, as in the case), then the diffraction from small d values can be recorded, resulting in a larger spanned q range.

Importantly, for the experiments presented in this paper, the fixed geometry of the ED experimental setup allowed a set of cradles to be allocated in the optical center of the diffractometer, enabling in-plane and out-of-plane rotations to be performed.

**Figure 2:**
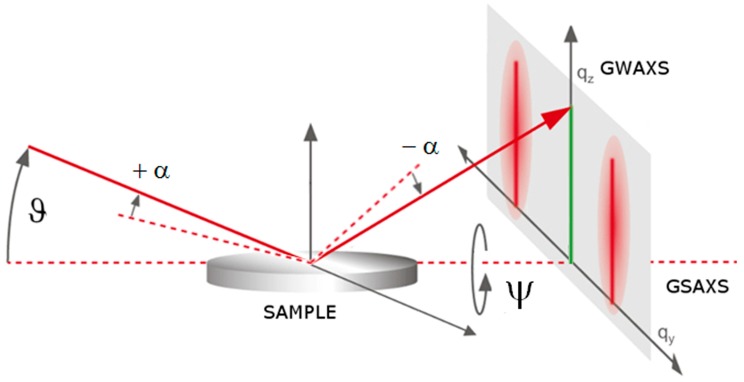
Schematic draft of energy dispersive GWAXS and GSAXS, where the X-ray scattering angle (ϑ), the in-plane α tilt, and out-of-plane Ψ tilt, as well as the relative q_y_ and q_z_ directions are shown.

In this way, since ED mode permits a multiple acquisition, the data collection, simultaneously as a function of the scattering angle ϑ, energy dispersive grazing wide-angle X-ray scattering (GWAXS), and grazing small-angle X-ray scattering (GSAXS) measurements was performed, tilting the sample along the in-plane direction (± α) and out-of-plane direction (Ψ), as shown in Figure 2. In this way, structural information along the z direction and along the x–y in-plane direction was obtained. A schematic draft, here, for GWAXS and GSAXS geometry, is shown in Figure 2.

**Figure 3:**
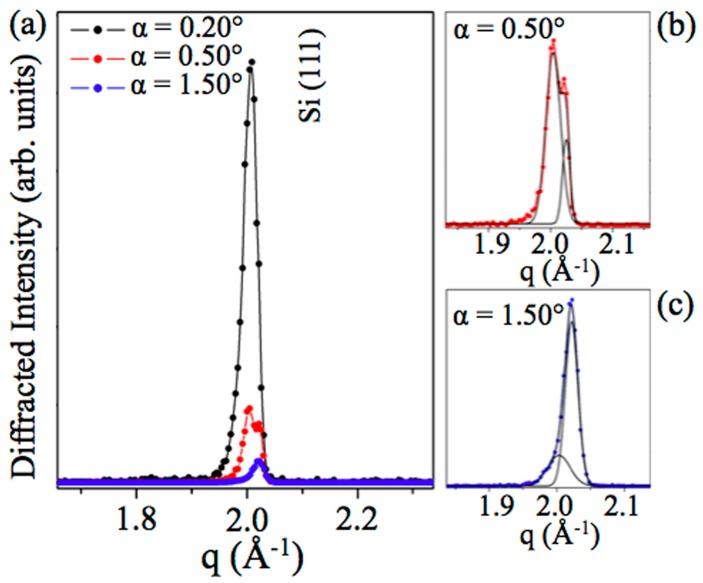
Energy dispersive out-of-plane rocking curves patterns from 10 MLs √3 × √3 Si film, grown on reconstructed Si(111)√3–Ag; (**a**) Si(111) diffraction peak (black dots) located at qz _Si(111)_ = 2.003 (5) Å^−1^; Si(111) and multilayer silicene diffraction peaks located at q_z 10 ML_ = 2.032 (5) Å^−1^ (red and blue dots). (**b**) and (**c**) are the expanded spectra from (a) with their Gaussian fits (black line).

At first, a rocking curve (RC) analysis was performed upon the 10 MLs √3 × √3 Si film grown on reconstructed Si(111)√3–Ag, moving symmetrically the sample along the ±α directions, thus keeping the whole scattering-angle unchanged (see Figure 2), to minimize the Si(111) contribution along the z direction and detect the out-of-plane reflection from 10 MLs √3 × √3 Si film signal more clearly. The results at different α-values are shown in Figure 3.

The Si(111) diffraction peak (black dots) was observed at q_z Si(111)_ = 2.003 (5) Å^−1^, corresponding to d_z Si(111)_ = 3.136 Å, matching literature [ICDD card Nr. 00-04-0783], and as the RC analysis was performed, interestingly, the presence of multilayer silicene diffraction peaks located at q_z 10 MLs_ = 2.032 (5) Å^−1^ became evident (red and blue dots) d_z 10 MLs_ = 3.092 (5) Å, in good agreement with reference [1,23]. In Figure 3b,c, the highly tilted EDXD patterns are highlighted, collected at α = 0.50° and 1.50°, respectively, in order to better evidence the coexistence of both crystalline signatures (Si(111) and 10 MLs silicene). The reported diffraction peak deconvolution was obtained by means of gaussian fits (black line). The Gaussians’ full width at half maximum are: FWHM _Si(111)_ = 0.023 Å^−1^; FWHM _10 MLs_ = 0.011 Å^−1^ at α = 0.50°; FWHM _Si(111)_ = 0.033 Å^−1^; FWHM _10 MLs_ = 0.019 Å^−1^ at α =1.50°.

**Figure 4:**
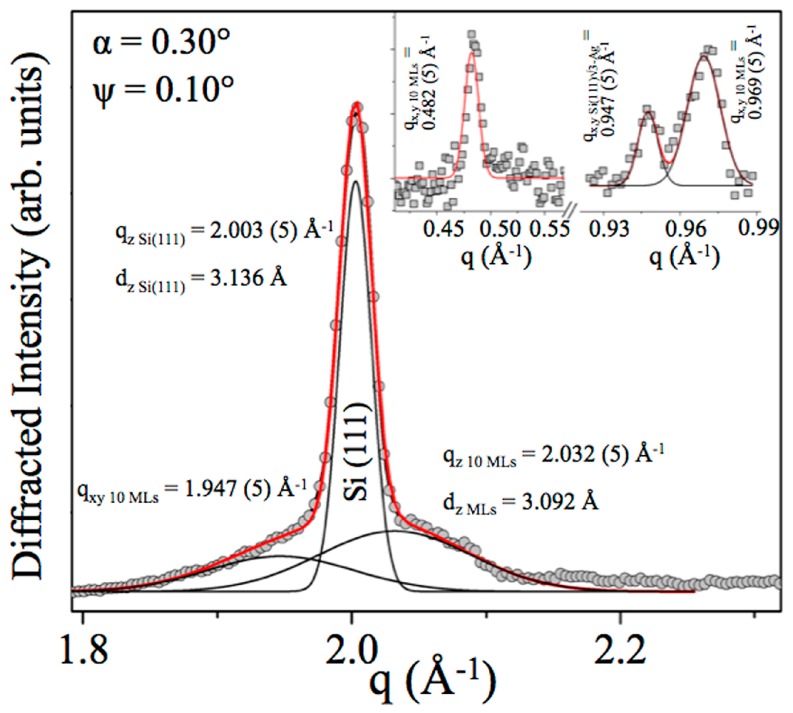
Energy dispersive GIXRD patterns from 10 MLs √3 × √3 Si film grown on reconstructed Si(111)√3-Ag: out-of-plane diffraction peaks located at q_z Si(111)_ = 2.003 (5) Å^−1^ and q_z 10 MLs_ = 2.032 (5) Å^−1^ with their Gaussian fits (black line) and in-plane second order reflection q_xy 10 MLs_ = 1.947 (5) Å^−1^. Peaks positions are the centroid of the Gaussians, and the full width at half maximum are: FWHM _Si(111)_ = 0.020 Å^−1^, FWHM _10 MLs_ = 0.120 Å^−1^ and FWHM_xy 10 MLs_ = 0.110 Å^−1^. Right inset is representative of the Si(111)√3–Ag reconstruction and 10 MLs √3 × √3 Si film, corresponding to the in-plane q_xy Si(111)√3-Ag_ = 0.947 (5) Å^−1^ (FWHM_xy Si(111)√3-Ag_ = 0.018 Å^−1^) and q_y 10 MLs_ = 0.969 (5) Å^−1^ (FWHM_xy 10 MLs_ = 0.030 Å^−1^). The left inset shows the in-plane-multiple order reflection q_xy 10 MLs_ = 0.482 (5) Å^−1^ (FWH_xy 10 MLs_ = 0.015 Å^−1^) from 10 MLs √3 × √3 Si film unit cell. The inset is adapted from Figure 6 of Ref. [1].

Subsequently, to deduce in-plane structural information, the cradle was tilted along the Ψ direction, thus partially projecting the X-ray momentum transfer on the film plane. In this way, tilting both α and Ψ, simultaneous acquisition of in-plane and out-of-plane structural features was gained, and the substrate contribution was minimised. Indeed, as visible in Figure 4, not only the previously described Si(111) and multilayer silicene diffraction peaks (q_z_) were observed, but the in-plane Si(111)√3–Ag reconstruction and 10 MLs √3 × √3 Si film in-plane reflections were detected.

Multilayer silicene in-plane second order reflection at q_xy 10 MLs_ = 1.947 (5) Å^−1^ was now visible, as well as the Si(111)√3-Ag reconstruction and 10 MLs √3 × √3 Si film, corresponding to the in-plane q_xy Si(111)√3-Ag_ = 0.947 (5) Å^−1^ and q_xy 10 MLs_ = 0.969 (5) Å^−1^ and plotted in the right inset. Furthermore, the left inset shows the in-plane multiple order reflection q_xy 10 MLs_ = 0.482 (5) Å^−1^ (FWHM_xy 10 MLs_ = 0.015 Å^−1^) from 10 MLs √3 × √3 Si film unit cell.

**Figure 5:**
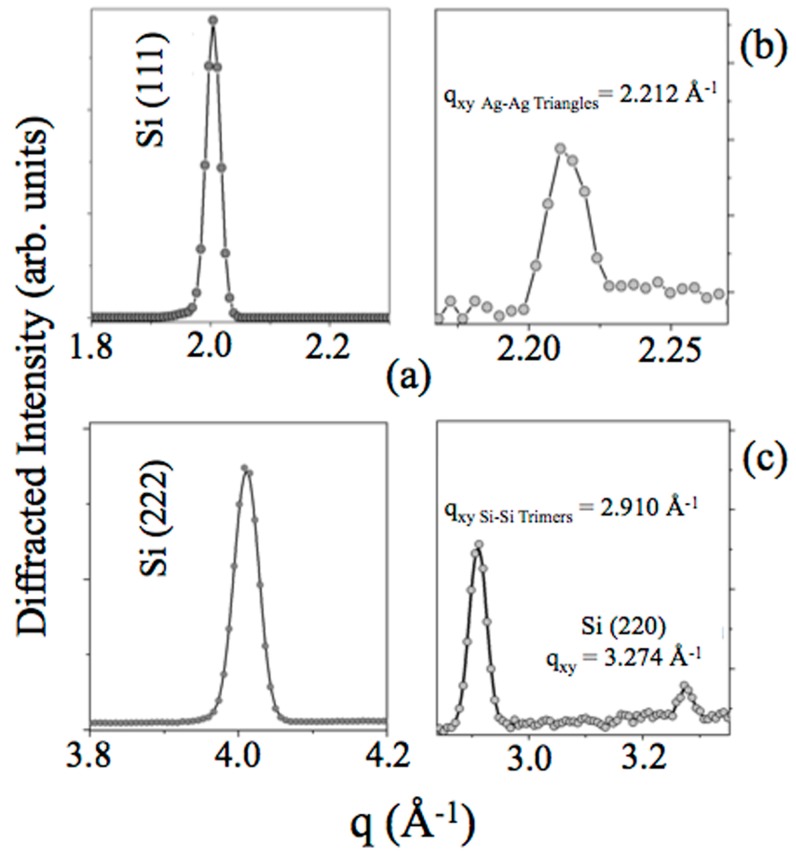
ED-GIXRD pattern of Si(111)√3–Ag substrate. (**a**) Si Diffraction peaks (q_z_) are labeled accordingly to JCPDS N. 27-1402. (**b**) The Ag–Ag triangles reflection at q_xy Ag-Ag Triangles_ = 2.212 (5) Å^−1^ is shown. (**c**) The Si(220) q_xy_ = (3.274 ± 0.005) Å^−1^ and the in-plane Si–Si trimers q_xy Si-Si Trimers_ = 2.91 Å^−1^ are highlighted.

The Si(111)√3-Ag template only was also characterized in order to observe out-of-plane and in-plane reflections of both Si and √3-Ag reconstruction, as an internal reference. As reported in Figure 5a, main reflections of the silicon substrate were detected in Bragg conditions, perfectly matching literature, as previously discussed [JCPDS Card Number: N. 27-1402].

Subsequently, to minimize the strong silicon contribution, rocking curve analysis was performed and ED-GIXRD pattern was collected, fixing α ~ 0.020° and ψ ~ 0.060°, respectively.

Figure 5b reports the Ag reflection at q_xy Ag-Ag Triangles_ = 2.212 (5) Å^−1^, corresponding to d_xy Ag-Ag Triangles_ = (2.840 ± 0.007) Å, and Figure 5c shows both q_xy Si-Si Trimers_ = (2.910 ± 0.005) Å^−1^, d_xy Si-Si Trimers_ = (2.159 ± 0.005) Å, related to the honeycomb-chained triangle (HTC) model of Si(111)√3–Ag [9,32,33,34] and the in-plane Si(111) q_xy Si(111)_ = 2.91 Å^−1^, d_220_ = (1.920 ± 0.003) Å, producing a lattice parameter of Si(111) = 3.842 Å, in agreement with the results reported in the reference [1].

In Table 1, we have summarized all ED-GIXRD-collected scattering vectors q_xy_ (in-plane)/q_z_ (out-of-plane) and the corresponding lattice parameters/vertical separations d from the Si(111) substrate, Si(111)√3–Ag template, and 10 MLs multilayer film, grown at ∼ 200 °C on the Si(111)√3–Ag template.

These results are in close agreement with the atomic distances between Ag triangles and Si trimers of the well-known HCT model for the √3-Ag reconstruction on Si(111) at room temperature [9,32,33,34] and those reported in reference [1], confirming the accuracy of the data.

**Figure 6:**
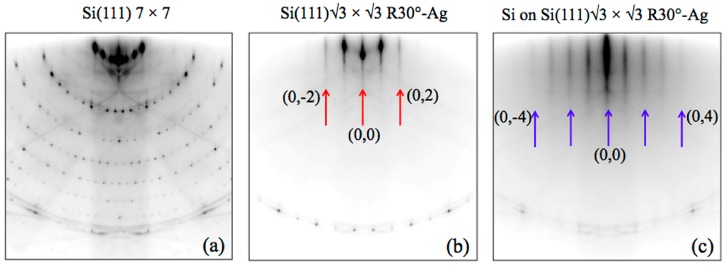
RHEED patterns collected at room temperature (RT) along the [11¯2] Si direction on clean Si(111) 7 × 7 (**a**), Si(111)√3 × √3 R30°–Ag interface (**b**), and after the deposition of 4 MLs of silicon, keeping the Si(111)√3–Ag template at ~200 °C. The red (blue) arrows in (**b**) and (**c**) indicate the (0,0) and the higher RHEED streaks order of √3-Ag and √3 × √3-Si.

The presence of √3 × √3 multilayer silicene reconstruction was also confirmed by the RHEED pattern, collected at RT (E_P_ = 18.6 keV) electron beam along the [11$\bar{2}$]. Figure 6 shows the typical RHEED patterns of clean Si(111) 7 × 7 (a) with, in addition, those recorded on the Si(111)√3–Ag interface (b) and after the growth of 4 MLs of silicon, grown at temperature of ~200 °C, just above a few degrees Celsius from getting an amorphous silicon film. The √3-Ag in (b) displays short RHEED streaks up to the second order (02,0–2), whereas in the √3 × √3 pattern after the Si deposition are clearly visible streaks until the fourth order (04,0-4).

**Figure 7:**
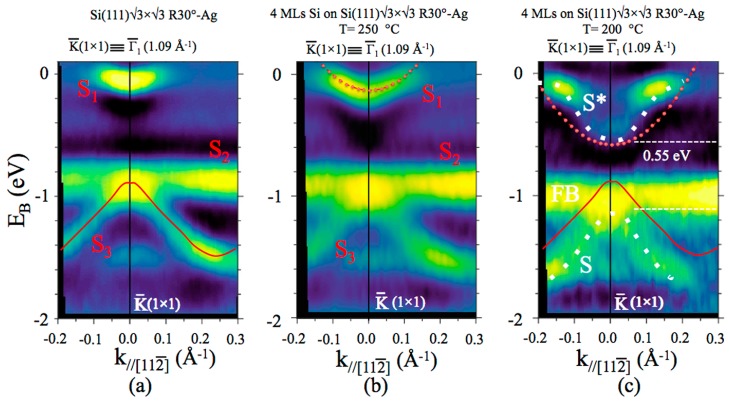
Second derivative band dispersion at RT from Si(111)√3 × √3 R30°–Ag (a); √3 × √3 of 4 MLs Si film grown at ~250 °C on Si(111)√3–Ag (**b**), and √3 × √3 Si film grown at ~200 °C on Si(111)√3 × √3–Ag (c). The origin of the wave vector is the $\bar{\Gamma }$_1_ point of the second Brillouin zone of the √3 × √3 SBZ at k = 1.09 Å^−1^ along the [11$\bar{2}$] direction. The three surface states S_1_, S_2_, and S_3_ belonging to the Si(111)√3–Ag interface are labeled, as well as S, S*, and FB from Si. The solid red line in (**a**), as well as the white dotted line in (**c**) are a guide for the eyes. Indeed, the dotted red curve is the best fit of the free-electron-like parabola of S_1_ in (**b**), superimposed to S* in (**c**).

Figure 7 shows the second derivative ARPES results taken at RT for pristine Si(111)√3–Ag interface (a); the growth of 4 MLs Si layers at high temperature of ~250 °C and low temperature: ~200 °C, (b) and (c), respectively. The band dispersions were collected in the second BZ at the high-symmetry point $\bar{\Gamma }$_1_, coincident with $\bar{K}$ (1 1), along the [11$\bar{2}$] direction.

**Figure 8:**
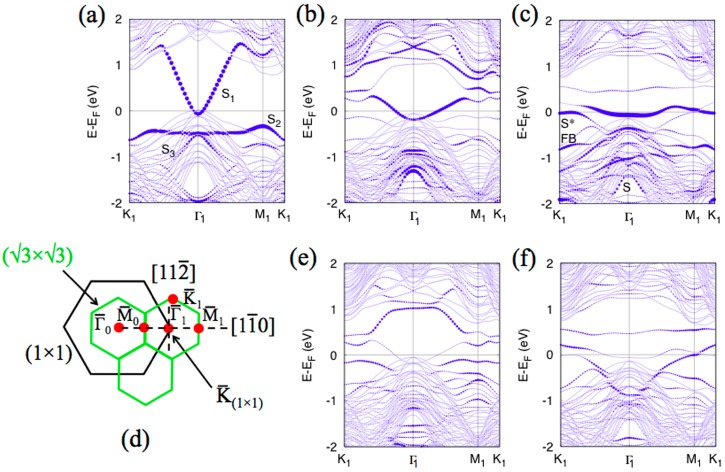
Calculated band structure of the bare √3 × √3 structure projected on Ag atoms (**a**), S_1_, S_2_, and S_3_ are marked; multilayer silicene model B1 projected on top Si layer (**b**) and on Ag (**e**); DP4 model projected on top Si layer (**c**), S*, FB, and S are labeled, and on Ag atoms (**f**). B1 and DP4 models are reported in reference [1]. Brillouin zones of √3 × √3 and (1 × 1) are shown in (**d**), as well as the $\bar{\Gamma }$_0_, $\bar{M}$_0_, $\bar{\Gamma }$_1_, $\bar{M}$_1_ high symmetry points, and [11$\bar{2}$], [1$\bar{1}0$] directions are indicated.

The bands dispersion at RT, reported in Figure 7a, showed three surface states: S_1_, S_2_, and S_3_, located at ~−0.2 and −0.9 eV below the Fermi level at $\bar{\Gamma }$_1_ are in good agreement with results of Si(111)√3–Ag, extensively explored in the past [35,36,37,38,39,40,41,42]. The S_1_ state, with a downward convex dispersion, the less dispersive S_2_, and the upward convex largely dispersing S_3_ were attributed, by first-principle calculation and scanning tunneling spectroscopy measurements, to surface states derived mainly from Ag 5*p*_x_ and 5*p*_y_ orbitals (S_1_), and states consisting mainly of Ag 5*s* orbitals with some Ag 5*p* contribution (S_2_ and S_3_) [43,44,45]. All these bands were well reproduced by present DFT calculations, as Figure 8a shows.

These characteristic surface states S_1_, S_2_, and S_3_ were observed in the dispersion of Figure 7b from Si film grown at ~250 °C on Si(111)√3–Ag, indicating that the Si layers consisted of similar electronic structures as those reported in (a), although the S_1_ surface state was slightly down-shifted in energy, most likely due to an extra doping from Ag atoms [37,46,47]. These findings are in nice agreement with results recently reported on the surfactant role of the Ag atoms in the growth of tetragonal Si layers on Si(111)√3–Ag upon the growth at temperature of ~250 °C [4].

However, a band similar to S_1_, albeit with slightly different dispersion, could also be obtained for certain multilayer silicene structures without on-top Ag atoms. Figure 8b and e show the calculated band structure for the lowest energy structure of 3 MLs silicone, described by the model B1 (3 MLs silicene) of Ref. [1]. Indeed, the band structure projected on the top Si layer and Ag layer, respectively, showed that the situation is more complicated and the presence of Ag atoms is not required to get the band structure reminiscent of the bare √3-Ag reconstruction. This finding sheds additional light on the long-standing discussion whether grown multilayer silicene is covered by Ag atoms or not [2,3,4,5,6,7,8]. Of course, this does not solve the problem, but certainly weakens arguments against uncovered multilayer silicene based on ARPES data.

Let us now examine the dispersion of the panel shown in Figure 7c from Si layers grown at lower temperature of about 200 °C. Here, we can easily observe that the old S_1_ and S_3_ structures disappeared, giving place to two new structures S and S*, located at ~−1.15 and ~−0.6 eV below the Fermi level, set at 0 eV, (separated by 0.55 eV) with a gap of ~0.6 eV at $\bar{\Gamma_{1}}$. While the nondispersive S_2_ band was replaced by a well-evident flat band (FB), even located at ~−1.0 eV. To better distinguish these bands, we have reported in Figure 7c the trace of S_1_ and S_3_ (in red) from panel (c) and traced the new dispersion of features S and S * with a dotted line (in white). It can be immediately noticed that both their shape and energy position are different from S_1_ and S_3_ bands, suggesting that they belong to different origins. In addition, we fit the S_1_ state in Figure 7b as a free-electron-like parabola (red dotted curve). In order to better show the relevant difference between S_1_, Ag-derived state and S*, Si-derived state, we superimpose this parabola to S*. The FB is also labeled.

We hypothesize that these extremely localized bands around $\bar{K}$ (1 × 1) belong to the 4 MLs multilayer silicene, although they are electronically affected by the presence of a confined two-dimensional electrons gas of the layer of Ag atoms trapped—quantum confined—between the silicon substrate and the silicene layers. This could explain the difference between these electronic structures and those, π and π* related to the Dirac cone, obtained from the epitaxial-grown silicene multilayer on single-crystal Ag(111) [15,48].

This hypothesis can be supported by the band structure calculated for the DP4 model for three-layer of silicene grown on Si(111)√3–Ag interface. This model predicts a layers stacking with internal honeycomb structure, showing a silicene √3 × √3 supercell on top of unperturbed Ag atoms. Clearly, band S* with similar intensity variations can be observed in Figure 8c,f. It looks like the top of the S* band is located at the Fermi level around point $\bar{K}$_1_ and disperses down until the $\bar{\Gamma_{1}}$ point. Moreover, the band features strong intensity variation. In fact, this is composed by two different bands: a flat band, around $\bar{K}$_1_, pinned to the Fermi level with Si 3*p*_z_ character and the dispersive band crossing the E_F_ due to Ag antibonding *d* orbitals (see Figure 8f). Since the Ag interface is covered by Si layers, the intensity of this Ag band measured by ARPES must be suppressed, leaving evident the new S* structure, Si related. This explains the apparent intensity variations. Furthermore, a flat band similar to S_2_ and labeled as FB in Figure 8c, also comes from the 3*p*_z_ orbitals of top Si atoms, according to the DP4 model. This band is located at lower energy, in near agreement with the ARPES data of Figure 7c. These Si-related bands, being almost flat and composed of *p*_z_ orbitals, are likely formed by the quantum size effect. Similar, the band S also comes from top Si atoms, but it has 3*p*_xy_ character. Note that we do not expect perfect agreement between DFT results and the ARPES spectra, since the calculations have been performed for the structure, which could have different number of Si layers than in the experiment. Nevertheless, some qualitative or even semiquantitative conclusions can be drawn. In particular, main features of the band structure, like for example surface- and interface-related states, should be present, independently of thickness of multilayer silicene. Therefore, we expect the proper atomic structure of multilayer silicene to have features of the DP4 model [1].

## 4. Conclusions

We reported new fingerprints of multilayer silicene grown on Si(111)√3 × √3 R30°–Ag at ~200 °C, by performing the energy-dispersive out-of-plane rocking curves and grazing incidence XRD patterns that clearly show the stacked distance between the silicene layers of 3.092 (5) Å and the first and multiple XRD orders of the √3 × √3 silicene unit cell size of 6.484 (5), both values different from tetragonal Si. Experimental angle-resolved photoemission spectroscopy on multilayer silicene evidences new electronic structures S, S*, and a flat band, FB, at $\bar{\Gamma_{1}}$, point, i. e. $\bar{K}$ (1 × 1) at 1.09 Å^−1^ in the second BZ along the [11$\bar{2}$] direction. They are separated by 0.55 eV and are similar to multilayer silicene grown on Ag(111) single crystal, whose characteristics are influenced by the 2D electrons gas of Ag atoms, confined between Si(111) substrate and multilayer silicene. Band-structure calculations performed on three honeycomb-stacked silicene layers confirm the ARPES results.

This work corroborates the results already presented in Ref. [1], through both new structural data and electronic properties, excluding the formation of ordinary tetragonal silicon and instead reinforcing the √3 × √3 multilayer silicene synthesis, grown at low temperature (~200 °C), on Si(111)√3–Ag template. 

## Figures and Tables

**Table 1:** **ED-GIXRD**.

Samples	Peak_xy_	q_xy_ (Å^−1^)	d_xy_ (Å) q	Peak_z_	q_z_ (Å^−1^)	d_z_ (Å)
**Si(111)**	(220)	3.274 (5)	1.919 (10)	(111)	2.003 (5)	3.136 (10)
				(220)	4.006 (5)	1.568 (10)
**Si(111)√3–Ag**	(d_Si-Si Trimers_)	2.212 (5)	2.840 (10)			
	(d_Ag-AgTriangles_)	2.910 (5)	2.159 (10)			
	d_Lattice parameter_	0.947 (5)	6.640 (10)			
**10 MLs silicene**	d_Lattice parameter_	0.969 (5)	6.484 (15)		2.032 (5)	3.092 (5)
		1.947 (5) 0.482 (5)	3.240 (10) 13.030(15) ^1^			

^1^ ED-GIXRD-collected scattering vectors q_xy_ (in-plane)/q_z_ (out-of-plane) and corresponding lattice parameters/vertical separations d from the Si(111) substrate, Si(111)√3–Ag template, and 10 MLs multilayer film, grown at ∼ 200 °C on the Si(111)√3–Ag template.
